# Supplementary material for: Constitutive activity of an atypical chemokine receptor revealed by inverse agonistic nanobodies
Source: Nat Commun. 2025 Dec 2;16:10828. doi: 10.1038/s41467-025-65858-x (PMC12673113; doi:10.1038/s41467-025-65858-x)
Supplement: Supplementary file 1 — Supplementary Information [file 41467_2025_65858_MOESM1_ESM.pdf]

Supplementary information for "Constitutive activity of an atypical chemokine receptor revealed by inverse agonistic nanobodies"

Supplementary information:

Supplementary Table 1. pEC50s and pIC50s (± SD) of CXCL12 and ACKR3-targeting Nbs for BRET experiments.

	**CXCL12 (Agonist)**	**VUN700 (Inverse Agonist)**	**VUN701 (Antagonist)**	**VUN702 (Inverse Agonist)**
**CXCL12 displacement**	9.9± 0.1	8.5 ± 0.1	8.7 ± 0.1	8.1 ± 0.2
**β-arrestin1 recruitment**	8.7 ± 0.2	8.2 ± 0.3	n.d.	8.1 ± 0.2
**β-arrestin2 recruitment**	8.9 ± 0.1	8.0 ± 0.2	n.d.	7.8 ± 0.1
**Internalization CAAX**	9.1 ± 0.1	8.1 ± 0.1	8.3 ± 0.2	7.5 ± 0.1
**Early endosomes Rab5a**	10.0 ± 0.2	7.0 ± 0.2	n.d.	7.2 ± 0.3
**Parental Intern. CAAX**	9.7 ± 0.2	8.3 ± 0.1	8.4 ± 0.1	8.1 ± 0.1
**dQ KO Intern. CAAX**	n.d.	8.2 ± 0.1	8.4 ± 0.1	8.0 ± 0.0
**Parental β-arrestin2 recruitment**	9.0 ± 0.3	n.d.*	n.d.	n.d.*
**dQ KO β-arrestin2 recruitment**	n.d.	n.d.	n.d.	n.d.
**Parental Intern. CAAX**	8.9 ± 0.1	7.8 ± 0.1	8.0 ± 0.2	7.4 ± 0.1

**β -arr2 KO Intern. CAAX**	9.0 ± 0.1	7.8 ± 0.1	8.2 ± 0.2	7.6 ± 0.1

n.d. (not determined), *window too small

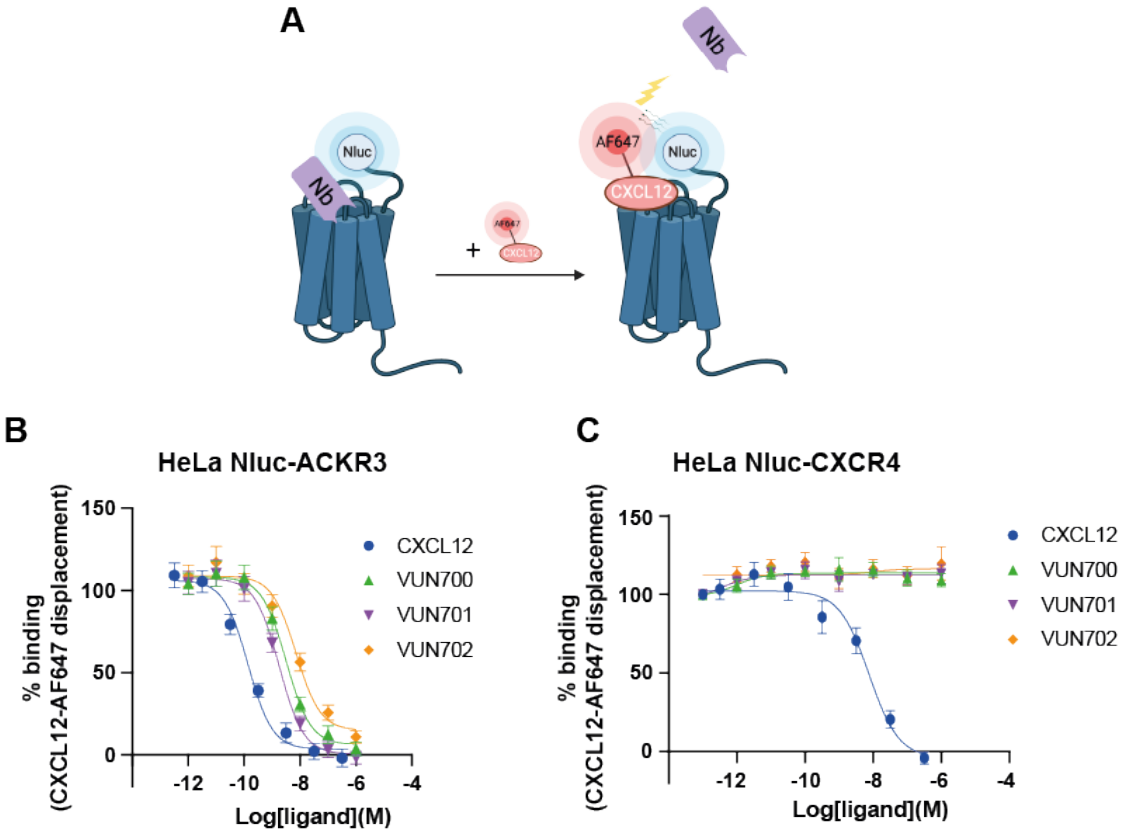

Supplementary Figure 1. All ACKR3-targeting nanobodies compete with CXCL12 to bind ACKR3, but not CXCR4. A) Schematic illustration of NanoBRET-based competition binding assay between ACKR3-targeting nanobodies and CXCL12-AF647. **B-C)** Competition curves of unlabeled CXCL12 (blue circle), or nanobodies VUN700 (green triangle), VUN701 (purple inverted triangle), or VUN702 (yellow diamond) by CXCL12-AF647 (3.3 nM) binding to Nanoluc-ACKR3 (B) or Nanoluc-CXCR4 (C) CRISPR Knock In HeLa cells. Data are shown as the average of three independent experiments performed in triplicate ± SD, normalized to maximum CXCL12-AF647 binding.

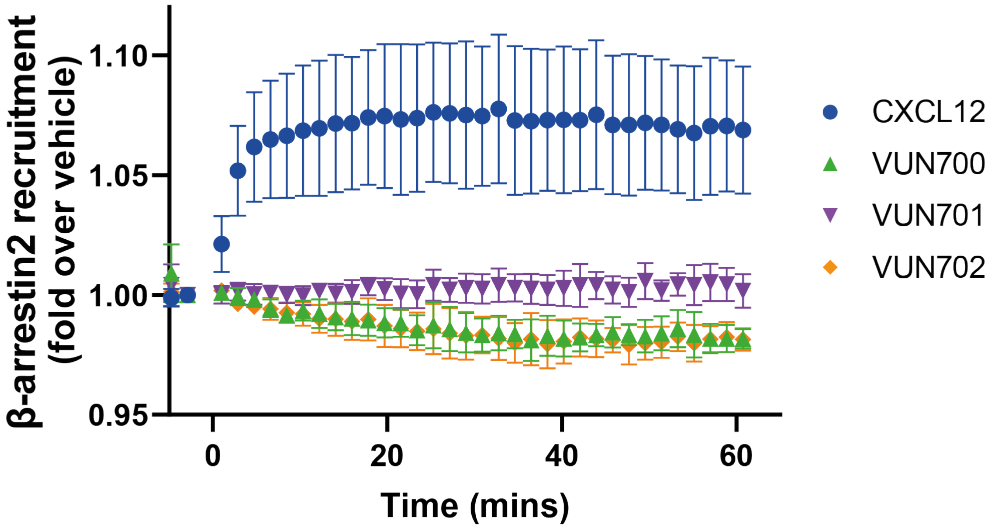

Supplementary Figure 2. ACKR3 nanobodies acts as inverse agonists and neutral modulators for β-arrestin2 recruitment in Chinese Hamster Ovary (CHO) cells. The time-dependent change in BRET over 60 min after treatment with either 316 nM of CXCL12 (blue circle) or 1 μM of VUN700 (green triangle), VUN701 (purple inverted triangle), or VUN702 (yellow diamond). Data is shown as the average ± SD of three independent experiments performed in triplicate.

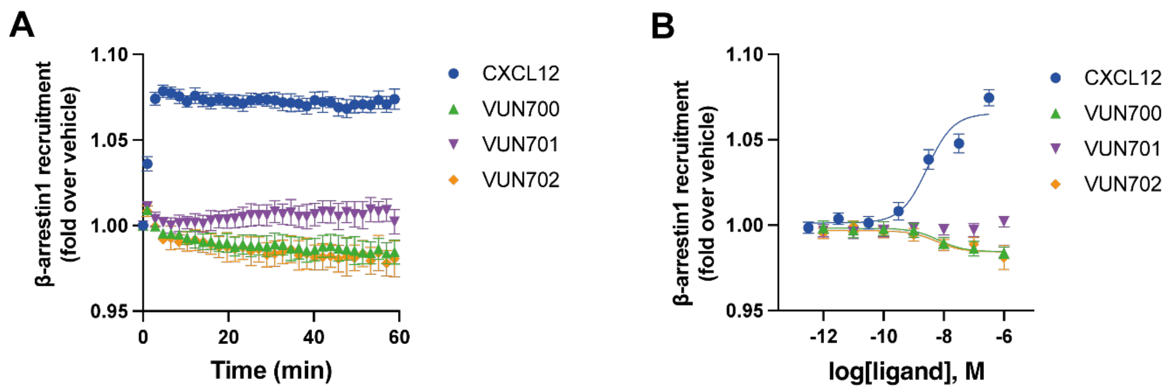

Supplementary Figure 3. ACKR3 nanobodies acts as inverse agonists and neutral modulators for β-arrestin1 recruitment. A-B) Recruitment of β-arrestin1-mVenus to ACKR3-Nluc measured by BRET. **(A)** The time-dependent change in BRET over 60 min after treatment with either 316 nM of CXCL12 (blue circle) or 1 μM of VUN700 (green triangle), VUN701 (purple inverted triangle), or VUN702 (yellow diamond). **(B)** Dose response curves of nanobodies or CXCL12 at 60 min, at 37 °C in HEK293T cells. Data is shown as the average ± SD of three independent experiments performed in duplicates.

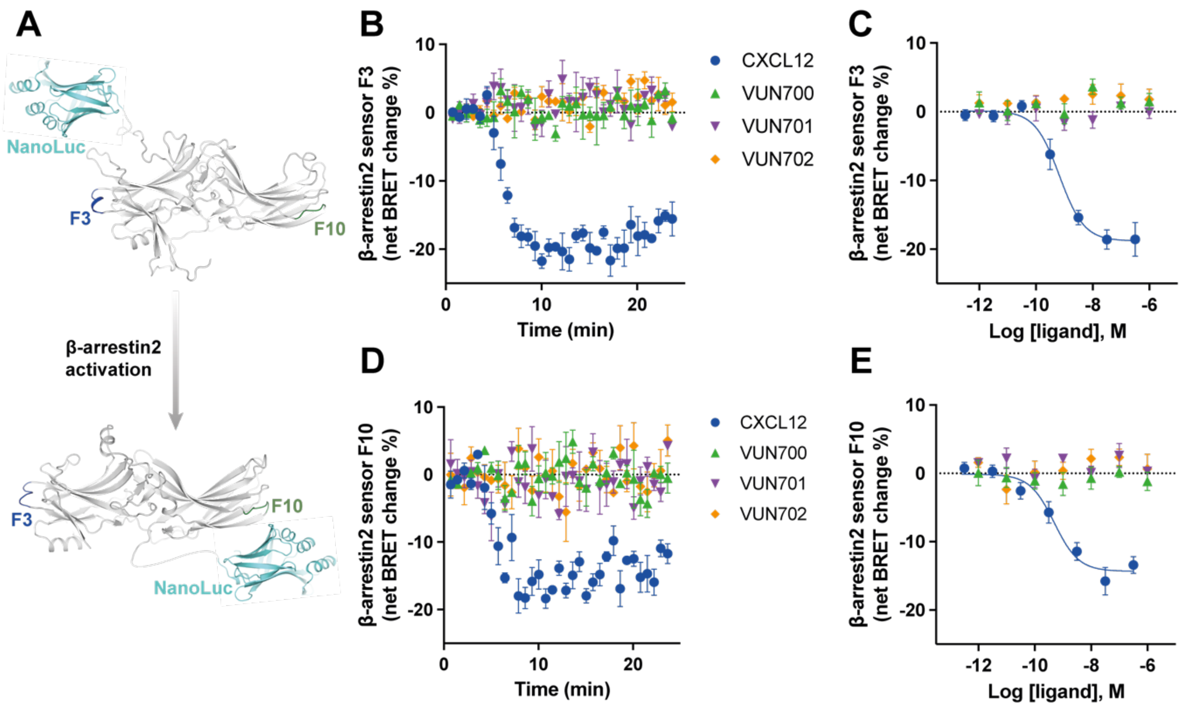

Supplementary Figure 4. Nanobody treatment of ACKR3 does not lead to a change in β-arrestin2 conformational biosensors F3 and F10 BRET. A) Schematic illustration of BRET-based FlAsH-tagged (CCPGCC) sensors F3 (between 48 and 49) and F10 (between 262 and 263) on β-arrestin2. **B-C)** F3 β-arrestin2 NanoBRET conformational biosensor with ACKR3 **(B)** time-dependent change in BRET over 25 min with either 316 nM of CXCL12 (blue circle) or 1 µM of VUN700 (green triangle), VUN701 (purple inverted triangle) or VUN702 (yellow diamond) and **(C)** DRCs effect of nanobodies at 25 min, at 37 °C in HEK293 cells. **D-E)** F10 β-arrestin2 NanoBRET conformational biosensor with ACKR3 **(D)** kinetics over 25 min with either 316 nM of CXCL12 or 1 µM of VUN700, VUN701 or VUN702 and **(E)** DRCs effect of nanobodies at 25 min, at 37 °C in HEK293 cells. Data is shown as the average ± SD of three independent experiments performed in triplicates.

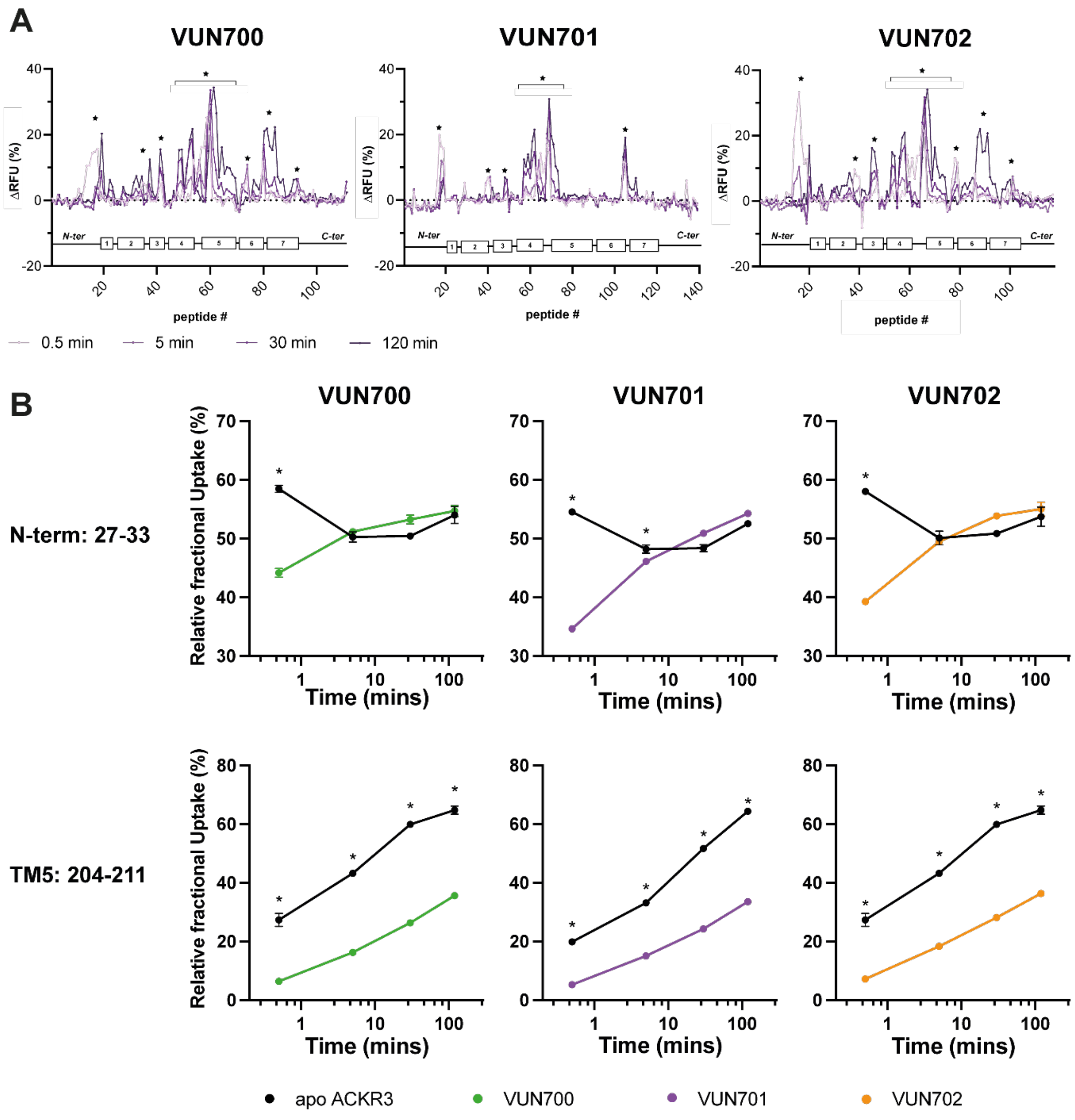

Supplementary Figure 5. HDX data of ACKR3 with nanobodies VUN700, VUN701, and VUN702. A) Butterfly plots showing time-dependent change in ΔRFU for identified ACKR3 peptides in both apo and bound forms. **B)** HDX uptake plots showing time-dependent change in relative fractional uptake for ACKR3 peptides at the N-terminal and TM5 levels upon nanobodies binding. Uptake represents the average and SD of three technical replicates from one biological preparation of ACKR3. Data is representative of three biological replicates. Statistically significant changes were determined using Deuteros 2.0 software[1] (*, $p \leq 0.01$).

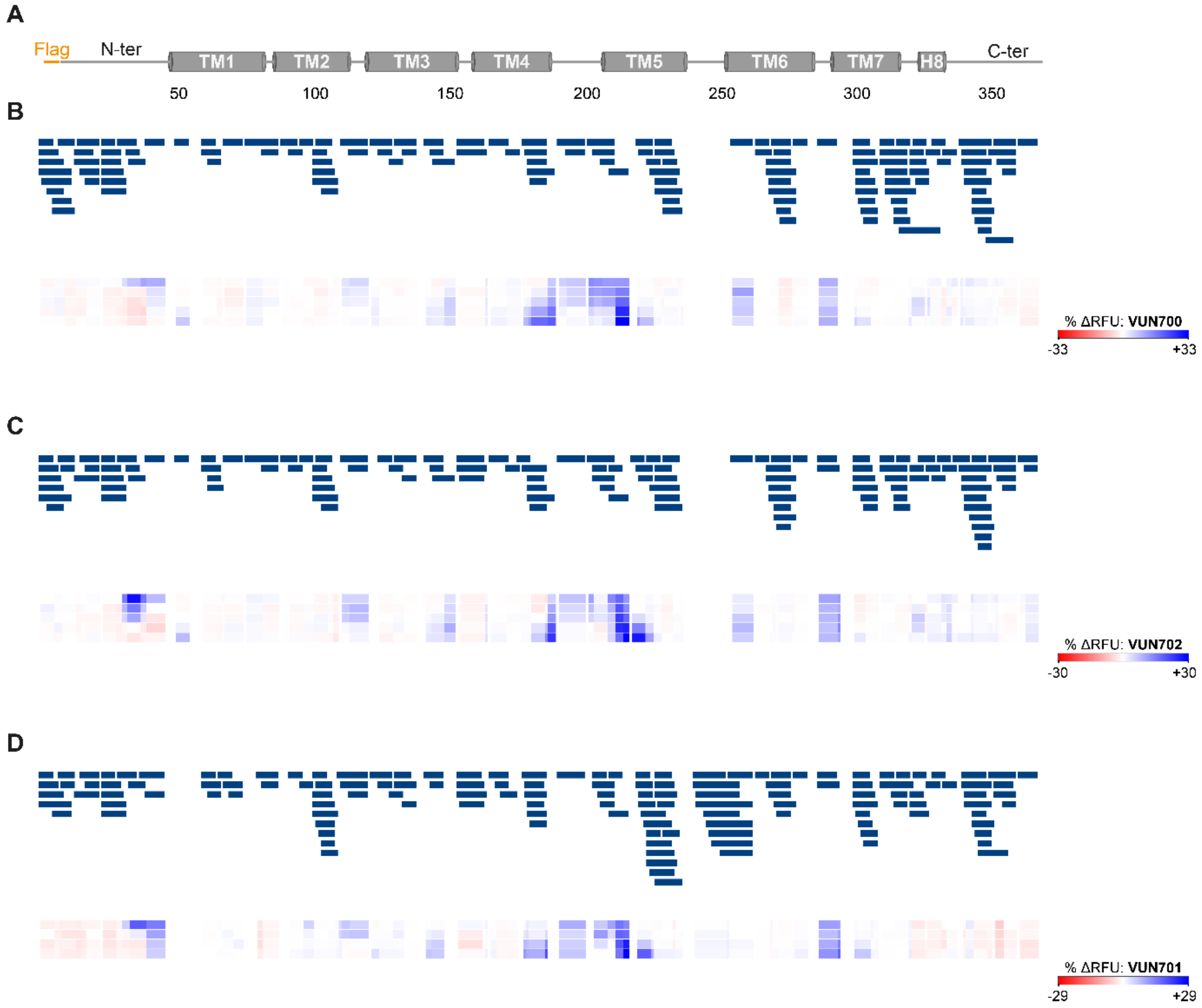

Supplemental Figure 6. Coverage maps and representative heat maps for tested nanobodies. A) Schematic representation of ACKR3 transmembrane helices in correspondence to residue numbers. **B-D)** Representative relative fractional uptake differences (%) and coverage maps for HDX analysis of ACKR3 in the presence of all tested nanobodies (apo – Nb-bound ACKR3). Deuteration was performed for 0.5, 5, 30 and 120 minutes for all states. For additional information, see supporting Data tables**.**

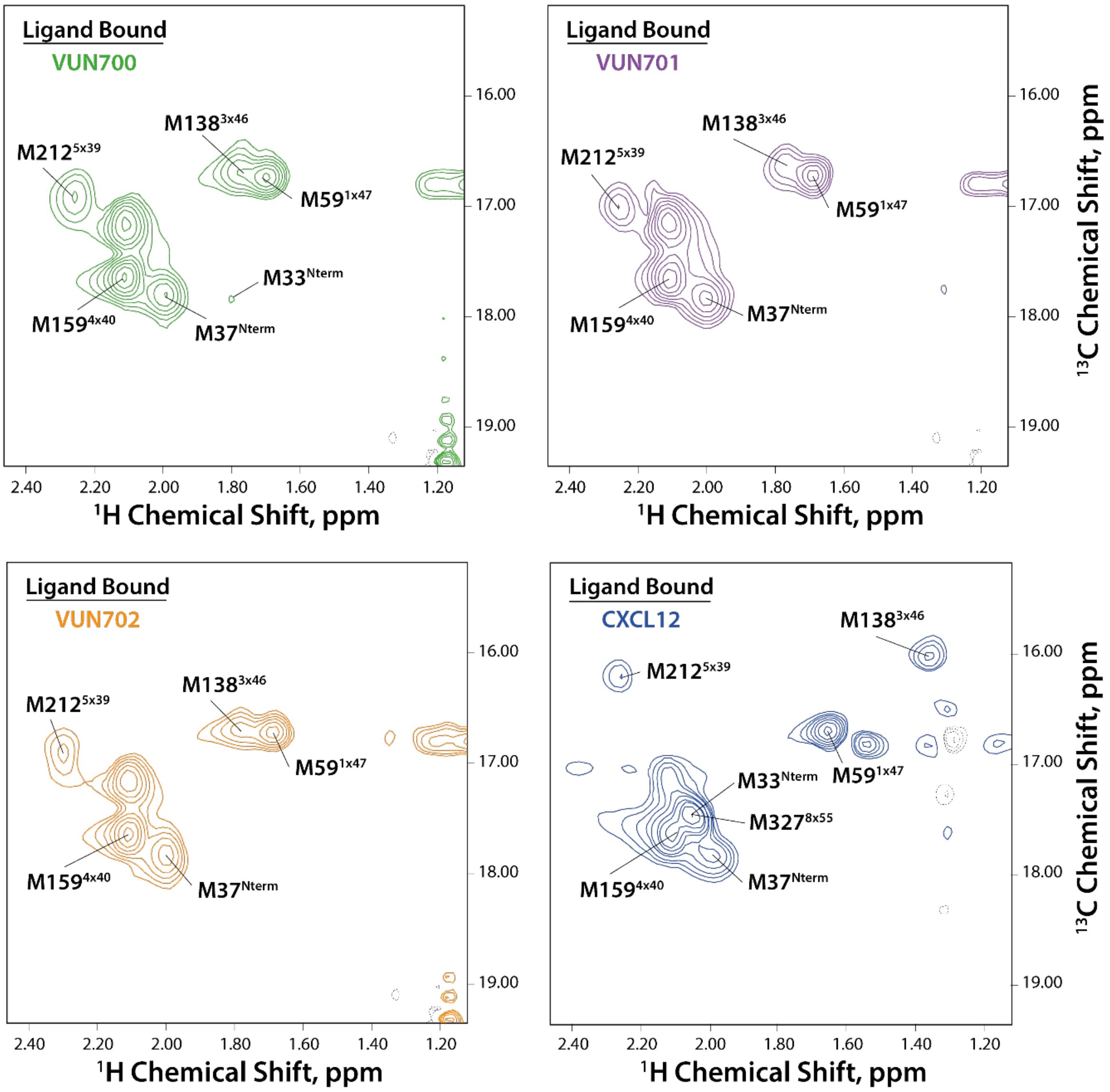

Supplementary Figure 7. NMR spectra of ACKR3 with nanobodies VUN700, VUN701, VUN702, and CXCL12. ^{1}H-^{13}C heteronuclear single quantum coherence NMR spectra of WT-ACKR3 with different ligands (three ACKR3-binding nanobodies and CXCL12[2]) at 310 K.

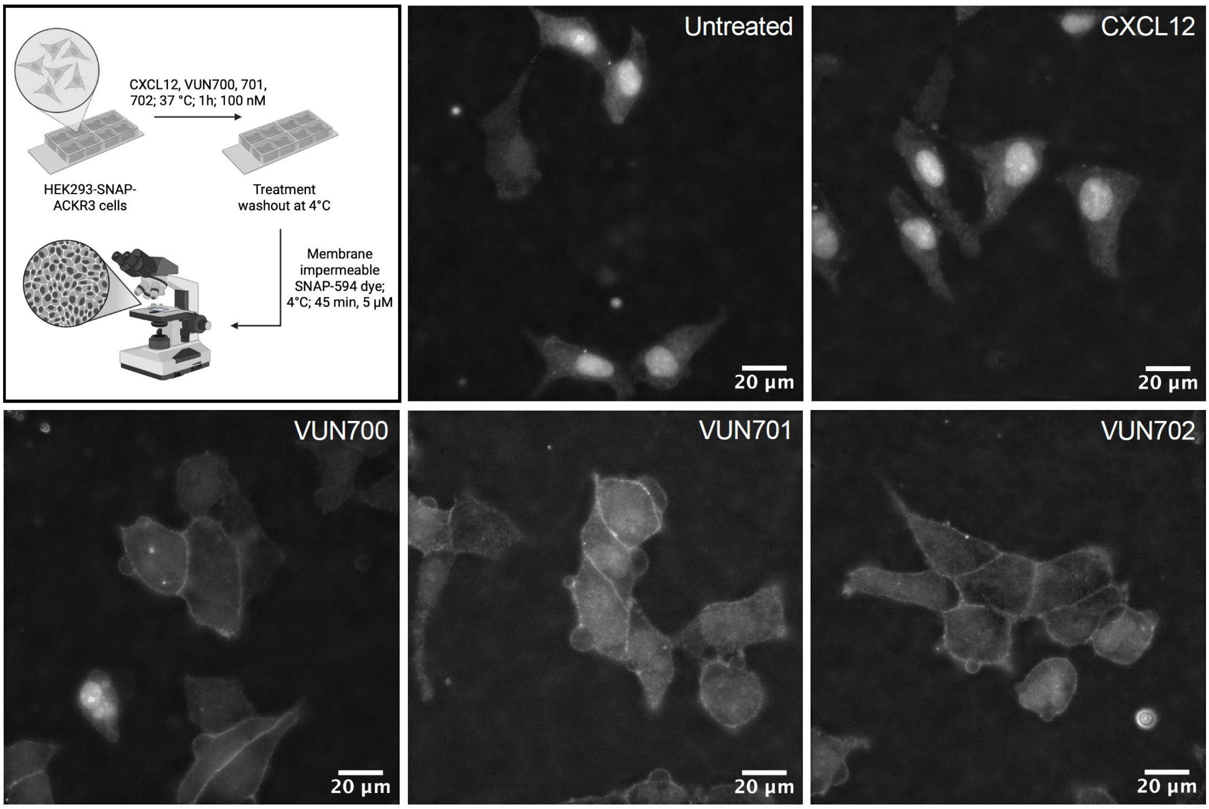

Supplementary Figure 8. ACKR3 nanobodies capture ACKR3 at the plasma membrane. Surface expression of ACKR3 detected by fluorescence microscopy in HEK293 cells stably expressing SNAP-ACKR3, labeled with cell-impermeable SNAP-Surface Alexa Fluor 594. The cells were treatment with 100nM of VUN700, VUN701, VUN702, or CXCL12 at 37 °C for 1 hour and ACKR3 as stained at 4 °C for 45 min using 5 μM of cell-impermeable SNAP-Surface Alexa Fluor 594.

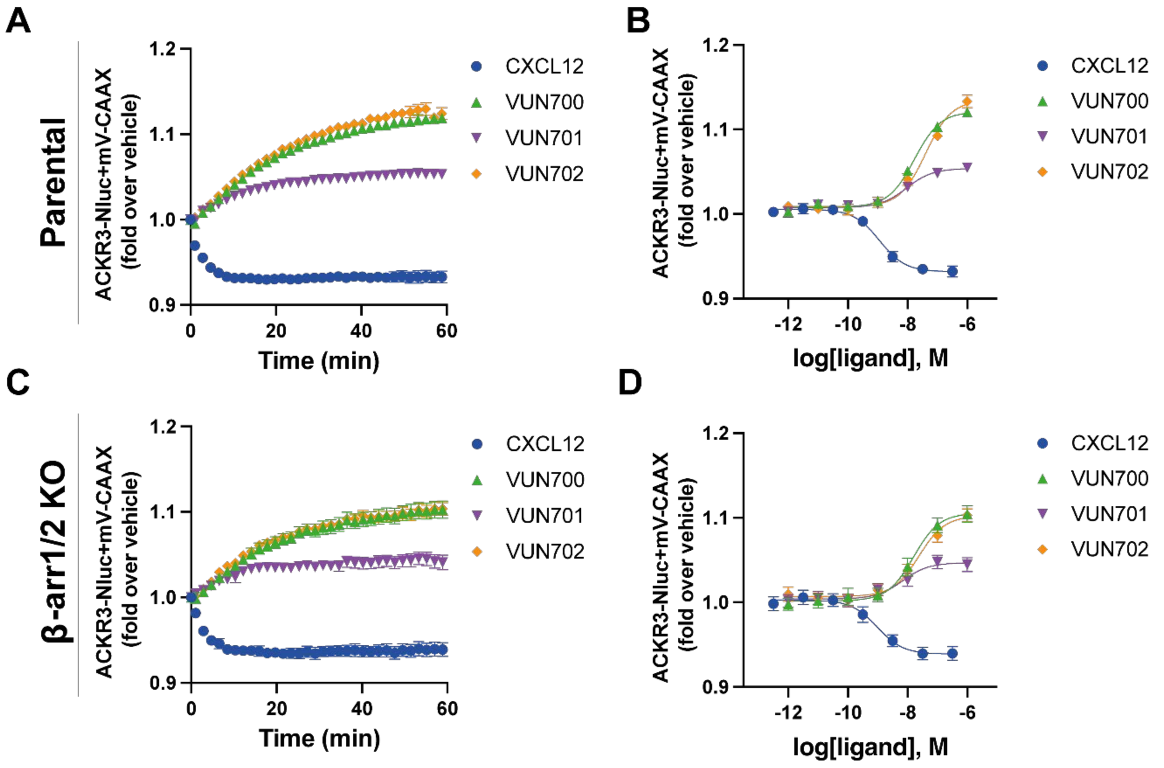

Supplementary Figure 9. ACKR3 nanobodies trap the receptor at the plasma membrane by a β-arrestin1/2 independent mechanism. A-D) Internalization measured by BRET in agonist mode, between donor ACKR3-Nluc and mV-CAAX in **(A, B)** HEK293 or in **(C, D)** HEK293 β-arrestin1/2 CRISPR KO cells. **(A, C)** Time-dependent change in BRET over 60 min with either 316 nM of CXCL12 (blue circle) or 1 µM of VUN700 (green triangle), VUN701 (purple inverted triangle), or VUN702 (yellow diamond) and **(B, D)** dose response curves of CXCL12 or nanobodies at 60 min, at 37 °C in HEK293T cells. Data is shown as the average ± SD of four independent experiments performed in triplicate.

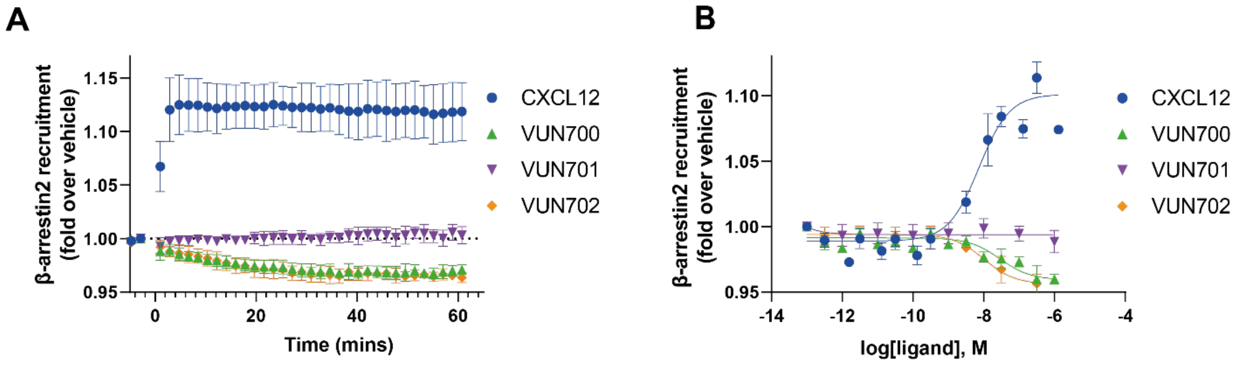

Supplementary Figure 10. ACKR3 nanobodies acts as inverse agonists and neutral modulators for β-arrestin2 recruitment in the presence of 10% FBS. Recruitment of β-arrestin2-mVenus to ACKR3-Nluc in the presence of 10% FBS, as measured by BRET. **(A)** The time-dependent change in BRET over 60 min after treatment with either 316 nM of CXCL12 (blue circle) or 1 µM of VUN700 (green triangle), VUN701 (purple inverted triangle), or VUN702 (yellow diamond). **(B)** Dose response curves of nanobodies or CXCL12 at 15 min, at 37 °C in HEK293T cells. Data is shown as the average ± SD of three independent experiments performed in triplicates.

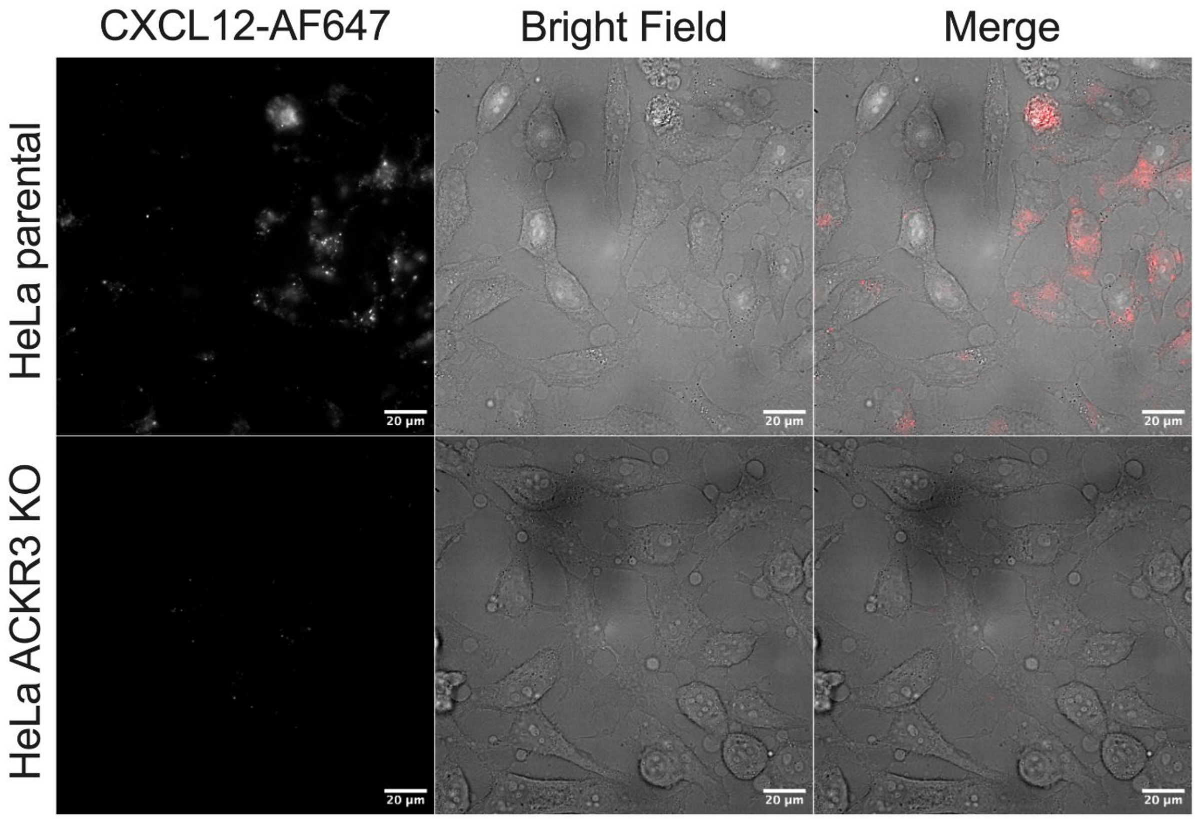

Supplementary Figure 11. HeLa ACKR3 KO cells show no CXCL12 uptake confirming the functional knockout of the atypical receptor. Uptake of CXCL12-AF647 by either HeLa parental or HeLa ACKR3 KO cells was detected by fluorescence microscopy. Cells were treated with 10 nM CXCL12-AF647 and 1 µM IT1t for 24 h before imaging. IT1t was included to prevent contributions of endogenous CXCR4.

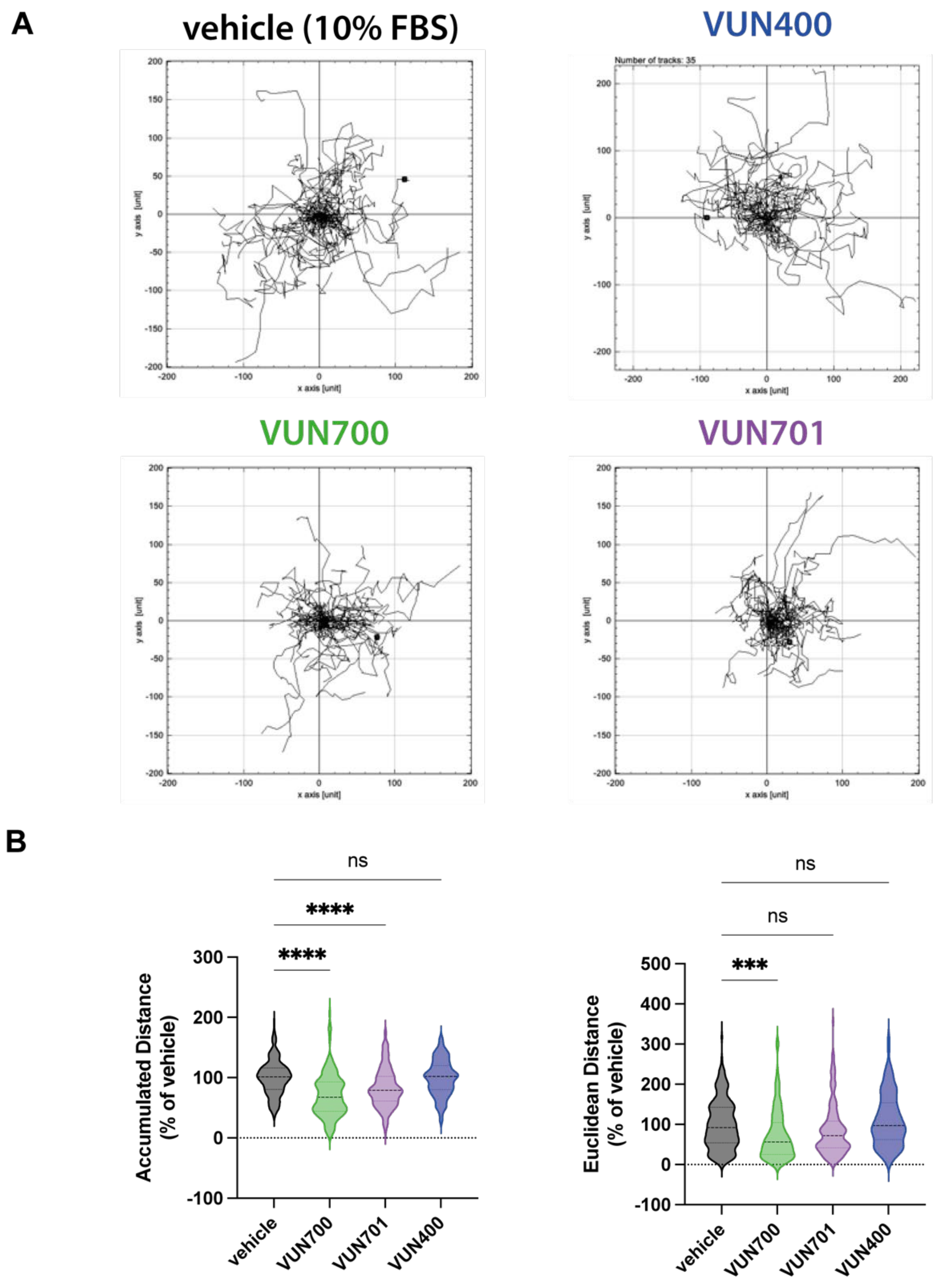

Supplemental Figure 12. Inverse agonistic ACKR3 nanobodies reduce basal motility of metastatic breast cancer cells . A) Representative basal motility paths of MDA-MB-231 metastatic breast cancer cells for 16 h in 10% FBS, vehicle condition (in black) or with 100 nM of VUN700 (in green), VUN701 (in purple) or control nanobody VUN400 (in blue). **B)** Accumulated distance (total distance traveled) and Euclidean distance (Start to end point distance) for individual cells from each of the conditions in **A.** The positions of at least 120

cells, selected across three replicates (~40 cells/repeat), were tracked and normalized to the vehicle condition. Significance was determined by one-way ANOVA Dunnett test ($p<0.0005$ (***), <0.0001 (****)).

Supplementary References

1 Lau, A. M., Claesen, J., Hansen, K. & Politis, A. Deuteros 2.0: peptide-level significance testing of data from hydrogen deuterium exchange mass spectrometry. *Bioinformatics* **37**, 270-272 (2021). https://doi.org:10.1093/bioinformatics/btaa677

2 Kleist, A. B. *et al.* Conformational selection guides β-arrestin recruitment at a biased G protein–coupled receptor. *Science* **377**, 222-228 (2022). https://doi.org:doi:10.1126/science.abj4922
