# Supplementary material for: Constitutive activity of an atypical chemokine receptor revealed by inverse agonistic nanobodies
Source: Nat Commun. 2025 Dec 2;16:10828. doi: 10.1038/s41467-025-65858-x (PMC12673113; doi:10.1038/s41467-025-65858-x)
Supplement: Supplementary file 2 — Description of Additional Supplementary Files [file 41467_2025_65858_MOESM2_ESM.pdf]

Description of Additional Supplementary Files

File name: Supplementary Data 1
Description: HDX summary tables.

File name: Supplementary Data 2
Description: HDX data tables.
