# Supplementary material for: Constitutive activity of an atypical chemokine receptor revealed by inverse agonistic nanobodies
Source: Nat Commun. 2025 Dec 2;16:10828. doi: 10.1038/s41467-025-65858-x (PMC12673113; doi:10.1038/s41467-025-65858-x)
Supplement: Supplementary file 5 — Reporting Summary [file 41467_2025_65858_MOESM5_ESM.pdf]

nature portfolio

Corresponding author(s): Martine J. Smit

Last updated by author(s): Jul 1, 2025

Reporting Summary

Nature Portfolio wishes to improve the reproducibility of the work that we publish. This form provides structure for consistency and transparency in reporting. For further information on Nature Portfolio policies, see our Editorial Policies and the Editorial Policy Checklist.

Statistics

For all statistical analyses, confirm that the following items are present in the figure legend, table legend, main text, or Methods section.

n/a	Confirmed	
☐	☒	The exact sample size (*n*) for each experimental group/condition, given as a discrete number and unit of measurement
☐	☒	A statement on whether measurements were taken from distinct samples or whether the same sample was measured repeatedly
☐	☒	The statistical test(s) used AND whether they are one- or two-sided<br>*Only common tests should be described solely by name; describe more complex techniques in the Methods section.*
☒	☐	A description of all covariates tested
☐	☒	A description of any assumptions or corrections, such as tests of normality and adjustment for multiple comparisons
☐	☒	A full description of the statistical parameters including central tendency (e.g. means) or other basic estimates (e.g. regression coefficient) AND variation (e.g. standard deviation) or associated estimates of uncertainty (e.g. confidence intervals)
☐	☒	For null hypothesis testing, the test statistic (e.g. *F*, *t*, *r*) with confidence intervals, effect sizes, degrees of freedom and *P* value noted<br>*Give P values as exact values whenever suitable.*
☒	☐	For Bayesian analysis, information on the choice of priors and Markov chain Monte Carlo settings
☒	☐	For hierarchical and complex designs, identification of the appropriate level for tests and full reporting of outcomes
☒	☐	Estimates of effect sizes (e.g. Cohen's *d*, Pearson's *r*), indicating how they were calculated

Our web collection on statistics for biologists contains articles on many of the points above.

Software and code

Policy information about availability of computer code

Data collection	Peptide Identification: ProteinLynx Global Server 3.0.3;<br>Peptide Filtering: DynamX 3.0;<br>NMR data collection: TopSpin 3.6;<br>Flow Cytometry: GuavaSoft 4.5.50;
Data analysis	Statistical analysis and curve fitting: GraphPad Prism 10.2.0;<br>HDX Statistical Analysis: Deuteros 2.0;<br>NMR Data Analysis: NMRPipe 9.6, XEASY 1.3.13;<br>Flow Cytometry: Floreada.io;<br>Image Processing: ImageJ/Fiji;<br>Single Cell Tracking: Chemotaxis and Migration Tool from Ibidi

For manuscripts utilizing custom algorithms or software that are central to the research but not yet described in published literature, software must be made available to editors and reviewers. We strongly encourage code deposition in a community repository (e.g. GitHub). See the Nature Portfolio guidelines for submitting code & software for further information.

Data

Policy information about availability of data

All manuscripts must include a data availability statement. This statement should provide the following information, where applicable:

- Accession codes, unique identifiers, or web links for publicly available datasets
- A description of any restrictions on data availability
- For clinical datasets or third party data, please ensure that the statement adheres to our policy

The HDX mass spectrometry data have been deposited to the ProteomeXchange Consortium via the PRIDE partner repository with the dataset identifiers PXD051149. All other data is contained within the manuscript.

Research involving human participants, their data, or biological material

Policy information about studies with human participants or human data. See also policy information about sex, gender (identity/presentation), and sexual orientation and race, ethnicity and racism.

Reporting on sex and gender	N/A
Reporting on race, ethnicity, or other socially relevant groupings	N/A
Population characteristics	N/A
Recruitment	N/A
Ethics oversight	N/A

Note that full information on the approval of the study protocol must also be provided in the manuscript.

Field-specific reporting

Please select the one below that is the best fit for your research. If you are not sure, read the appropriate sections before making your selection.

☒ Life sciences ☐ Behavioural & social sciences ☐ Ecological, evolutionary & environmental sciences

For a reference copy of the document with all sections, see nature.com/documents/nr-reporting-summary-flat.pdf

Life sciences study design

All studies must disclose on these points even when the disclosure is negative.

Sample size	Assay replicates were chosen to demonstrate reproducibility and enable statistically significant results. No sample size calculations were performed. A sample size of at least three was used for all statistical analysis to conform with the standard in the field of molecular pharmacology.
Data exclusions	No data was excluded from analysis.
Replication	Experiments were performed at least three independent times. Positive controls were included with BRET experiments and occasional replicates were performed to confirm data.
Randomization	Randomization was not relevant to this study. The results were not subject to investigator bias and no patients were used in this study.
Blinding	The investigators were not blinded during data collection or analysis. The data collected are not subject to investigator bias.

Reporting for specific materials, systems and methods

We require information from authors about some types of materials, experimental systems and methods used in many studies. Here, indicate whether each material, system or method listed is relevant to your study. If you are not sure if a list item applies to your research, read the appropriate section before selecting a response.

Materials & experimental systems

n/a	Involved in the study
☐	☒ Antibodies
☐	☒ Eukaryotic cell lines
☒	☐ Palaeontology and archaeology
☒	☐ Animals and other organisms
☒	☐ Clinical data
☒	☐ Dual use research of concern
☒	☐ Plants

Methods

n/a	Involved in the study
☒	☐ ChIP-seq
☒	☐ Flow cytometry
☒	☐ MRI-based neuroimaging

Antibodies

Antibodies used: 11G8-PE, #FAB4227 R&D Systems, 10ul/one million cells

Validation: Per manufacturer's website, "Detects human CXCR7/RDC-1 in flow cytometry of five distinct human CXCR7 transfectants, but not their respective parental lines."

Eukaryotic cell lines

Policy information about cell lines and Sex and Gender in Research

Cell line source(s):
Human Embryonic Kidney Cells (HEK) 293T were obtained from ATCC;
Chinese Hamster Ovary cells were obtained from ATCC;
HeLa cells were obtained from ATCC;
MDA-MB-231 cells were obtained from ATCC;
HEK293 b-arrestin1/2 KO cells and corresponding parental cells were obtained from Asuka Inoue (Tohoku University);
HEK293 GRK KO cells and corresponding parental cell were obtained from Carsten Hoffmann (University Hospital Jena);
NanoLuc-CXCR4 and NanoLuc-ACKR3 CRISPR Knock In HeLa cells were obtained from Stephen Hill (University of Nottingham);
Stable HEK293 expressing SNAP-tagged ACKR3 were obtained from Meritxel Canals (University of Nottingham);
ACKR3 KO HeLa cells were produced in this study (Vrije Universiteit Amsterdam)

Authentication: ACKR3 KO HeLa cells were confirmed by genomic sequencing and functional characterization of the receptor knockout by detection of CXCL12 uptake.

Mycoplasma contamination: Cells were tested regularly for mycoplasma contamination.

Commonly misidentified lines (See ICLAC register): No commonly misidentified cell lines were used in this study.

Plants

Seed stocks: N/A

Novel plant genotypes: N/A

Authentication: N/A
